# Identification of genes influencing the evolution of *Escherichia coli* ST372 in dogs and humans

**DOI:** 10.1099/mgen.0.000930

**Published:** 2023-02-08

**Authors:** Paarthiphan Elankumuran, Glenn F. Browning, Marc S. Marenda, Amanda Kidsley, Marwan Osman, Marisa Haenni, James R. Johnson, Darren J. Trott, Cameron J. Reid, Steven P. Djordjevic

**Affiliations:** ^1^​ Australian Institute for Microbiology and Infection, School of Life Sciences, Faculty of Science, University of Technology Sydney, Ultimo, NSW, Australia; ^2^​ Asia-Pacific Centre for Animal Health, Department of Veterinary Biosciences, Melbourne Veterinary School, Faculty of Veterinary and Agricultural Sciences, University of Melbourne, Parkville and Werribee, Victoria, Australia; ^3^​ Australian Centre for Antimicrobial Resistance Ecology, School of Animal and Veterinary Sciences, University of Adelaide, Roseworthy, Australia; ^4^​ Laboratoire Microbiologie Santé et Environnement, Doctoral School of Sciences and Technology, Faculty of Public Health, Lebanese University, Tripoli, Lebanon; ^5^​ Department of Public and Ecosystem Health, College of Veterinary Medicine, Cornell University, Ithaca, NY, USA; ^6^​ ANSES, Université de Lyon, Unité Antibiorésistance et Virulence Bactériennes, Lyon, France; ^7^​ Minneapolis VA Medical Center, Minneapolis, MN, USA

**Keywords:** canine, *E. coli*, ExPEC, genomic epidemiology, pathogen evolution, *pdu *operon, ST372

## Abstract

ST372 are widely reported as the major *Escherichia coli* sequence type in dogs globally. They are also a sporadic cause of extraintestinal infections in humans. Despite this, it is unknown whether ST372 strains from dogs and humans represent shared or distinct populations. Furthermore, little is known about genomic traits that might explain the prominence of ST372 in dogs or presence in humans. To address this, we applied a variety of bioinformatics analyses to a global collection of 407 ST372 *E. coli* whole-genome sequences to characterize their epidemiological features, population structure and associated accessory genomes. We confirm that dogs are the dominant host of ST372 and that clusters within the population structure exhibit distinctive O:H types. One phylogenetic cluster, ‘cluster M', comprised almost half of the sequences and showed the divergence of two human-restricted clades that carried different O:H types to the remainder of the cluster. We also present evidence supporting transmission between dogs and humans within different clusters of the phylogeny, including M. We show that multiple acquisitions of the *pdu* propanediol utilization operon have occurred in clusters dominated by isolates of canine source, possibly linked to diet, whereas loss of the *pdu* operon and acquisition of K antigen virulence genes characterize human-restricted lineages.

## Data Summary

All genomes described for the first time in this article are publicly available and were deposited in the National Center for Biotechnology Information (NCBI) Sequence Read Archive (SRA) under BioProjects PRJNA678027 and PRJNA827950. Individual SRA, BioSample and BioProject accession numbers for all sequences used in the study can be found in Table S1. The data analysis and visualization scripts are also available at https://github.com/CJREID/ST372 and can be used to reproduce all data analysis.

**Impact Statement**

This work is significant because it advances understanding of the epidemiology, population structure and evolution of *Escherichia coli* ST372 – the dominant type of *E. coli* causing infections in dogs and an emerging cause of human infections. We identified several key genes that are associated with the expansion of ST372 *E. coli* and argue that both dogs and humans have influenced their evolution. Intriguingly, genes encoding metabolism of common dog food additives were strongly associated with the major subgroup of ST372, suggesting a role for diet in the evolution of this pathogen. Our work supports consideration of the close relationship between humans and dogs when studying infectious organisms that affect both. Furthermore, it shows the value of genomic epidemiological studies for identification of genomic factors associated with pathogen evolution that can drive new hypotheses for experimental work.

## Introduction

*Escherichia coli* is the most frequently isolated Gram-negative pathogen globally. Over 11 000 *E. coli* multilocus sequence types (STs) (MLSTs) are reported in Enterobase, but only 20 of these are estimated to be responsible for more than 85 % of *E. coli* extraintestinal infections [1]. Despite debate surrounding molecular and source-based definitions, extraintestinal pathogenic *E. coli* (ExPEC) can be practically defined as *E. coli* isolated from an infected extraintestinal site, although this is not always a guarantee of classical ExPEC status defined by molecular typing [2]. Although MLST remains a gold standard of typing for *E. coli *, whole-genome sequencing (WGS) has revealed significant diversity below the ST level. Numerous pandemic *E. coli* STs can be divided into lineages exhibiting distinct genomic characteristics, often driven by mobile genetic elements (MGEs), in conjunction with diverse ecological and host associations [3–7]. Understanding how these lineages evolve within a particular host or niche and why particular MGEs persist within lineages could inform the development of mitigation strategies to combat the ongoing global issue of ExPEC [1].

The role of MGEs, such as F virulence plasmids, genomic islands and phage-related elements in the evolution of *E. coli* lineages, and in determining their host range, is an area of active study [4, 6, 8, 9]. F plasmids such as pUTI89-like and ColV-like plasmids are important contributors to fitness and virulence that carry genes involved in iron-acquisition [10, 11]. However, they have differential distributions in terms of their presence in animal and human hosts, *E. coli* STs and sub-ST lineages. For example, pUTI89-like plasmids are almost entirely restricted to humans and are known to associate with specific lineages within ExPEC-associated STs, such as ST131 and ST95 [4, 5]. By contrast, ColV plasmids are highly prevalent within *E. coli* from poultry and pigs, and in a broader range of *E. coli* STs, but also occur in some *E. coli* isolates from human infections [6]. Beyond plasmids, the role of genomic islands and phages in the evolution and pathogenicity of ExPEC has been described in detail, but little is known regarding their distribution among non-human hosts and *E. coli* lineages [12, 13]. The major implication of these findings is that the evolution and epidemiology of ExPEC should be regarded in terms of a complex web of interactions between *E. coli* lineages at varying scales – including phylogroups, STs and ST sub-lineages – and their MGEs – against a backdrop of ongoing selection via a multiplicity of niches within diverse hosts and environments. This conception is critical in the quest to gain a holistic understanding of ExPEC evolution and epidemiology.

Due to the increased acknowledgement of the role of non-human (though often human-impacted) sources in a proportion of human ExPEC infections, the historically anthropocentric nature of ExPEC genomic epidemiology has moved towards a One Health perspective. Companion animals, particularly dogs, have also received attention, acknowledging the close interaction between humans and their pet dogs, and, by extension, the risk of sharing pathogens between them. Urinary tract infections (UTIs) are one of the primary reasons for dog owners to consult a veterinarian, and approximately 14 % of dogs will experience at least one bacterial UTI during their lifetime [14]. Contact with dogs is a noted risk factor for human acquisition of ExPEC [15] and prominent lineages associated with ExPEC, such as ST73, ST12, ST127 and ST131 have been isolated from the urine of dogs with UTIs as well as faecal samples from healthy dogs [1, 16–20]. Whole-genome sequencing studies also show that some STs, and closely related strains within these STs, are carried by both dogs and humans [16, 21–23]. Carriage of genes encoding resistance to clinically important antibiotics is also reported [24–26].

These data might give the impression that canine ExPEC are simply a subset of human ExPEC, present due to repetitive human-to-canine transfer, but the dominant ExPEC among dogs, ST372, is comparatively uncommon in humans. Multiple studies have identified ST372 as the most prevalent ST in dogs and although ST372 have been identified in human infections, their apparent frequency compared to other STs in humans is low [1]. Several studies have suggested that ST372 may be a zoonotic pathogen and others have presented evidence of host sharing of *E. coli* ST372 causing clinical UTI [19]. Recent work by Flament-Simon *et al*. showed that O:H types O83:H31 and O18:H31 were associated with human source ST372, whereas O4:H31 and O15:H31 were associated with canine source isolates, implicating O:H type as a potential factor in adaptation to each host [16]. Beyond dogs and humans, *E. coli* ST372 have also been identified globally in diverse wildlife, including migratory birds and fruit bats, wastewater, livestock, drinking water and wetlands [27–32]. To the best of our knowledge, a large-scale genomic epidemiological study synthesizing host distribution and defining a clear population structure and the characteristics of different sub-lineages in ST372 is yet to be performed. To address this, we assembled a global collection of 407 *E. coli* ST372 whole-genome sequences from a wide variety of sources, defined their population structure and O:H types, identified genomic linkage between epidemiologically unrelated isolates, and performed a pan-genome-wide association study to identify acquired genes associated with different lineages.

## Methods

### The genome collection

Genome sequences belonging to 407 strains of *E. coli* ST372 of diverse epidemiological origin were used here. Of these, 285 sequences were obtained from Enterobase (http://enterobase.warwick.ac.uk), an online database housing enteric bacterial genomes (accessed on 11 January 2021). Initially, the database was queried for *E. coli* belonging to ST372 and a summary spreadsheet with the corresponding accession numbers and relevant metadata was downloaded. This data was filtered to exclude strains without sound accession numbers, source details and continent of origin. The final list of filtered samples was used to query the National Center for Biotechnology Information (NCBI), European Bioinformatics Institute (EBI) and DNA Data Bank of Japan sequence read databases. Sequence reads for all strains were downloaded with parallel-fastq-dump (https://github.com/rvalieris/parallel-fastq-dump). These 285 genome sequences were named using their NCBI, EBI or DDBJ accession numbers, corresponding to SRR, ERR or DRR prefixes, respectively.

Sixty-eight of the genome sequences used were from a collection of 399 canine-origin *E. coli* strains obtained from the Melbourne Veterinary School, University of Melbourne, Melbourne, Australia described previously [33]. The strains were sequenced at the University of Technology sequencing facility. These strains carry a ‘MVC_’ (meaning ‘Melbourne Veterinary Collection’) prefix followed by a one to three-digit numeral specifying individual strains from the collection. Twenty-eight of the additional sequences were obtained from isolates of human clinical and commensal *E. coli* administered by the Minneapolis Veterans Association Medical Center Hospital, Minnesota, USA. These sequences carry the prefix MVAST or CVAST followed by numerals indicating the individual isolates. Samples were received as culture swabs. A further 18 genome sequences, primarily originating from canine UTIs, were obtained from collaborators at the Anses laboratory in Lyon. Samples were received as raw sequence reads in fastq format. The remaining eight sequences originate from a larger collection of *E. coli* isolates from colorectal swabs of silver gull (*Chroicocephalus novaehollandiae*) from Five Islands, NSW, Australia. These strains are named with the prefix ‘SD’. Apart from sequences received as fastq files, all sequences were generated as described below.

### DNA sequencing

Sample swabs were streaked onto lysogeny broth (LB) agar plates and single colonies collected for culture in 10 ml liquid LB. Following overnight culture, total cellular DNA was extracted using the ISOLATE II Genomic DNA (Bioline) kit following the manufacturer’s standard protocol for bacterial cells and was stored at 4 °C. Library preparation was done by the Australian Institute for Microbiology and Infection Core Sequencing Facility at the University of Technology Sydney, following the adapted Nextera Flex library preparation kit process, Hackflex [34]. Briefly, genomic DNA was assessed quantitatively using the Quant-iT PicoGreen dsDNA assay kit (Invitrogen, USA). Each sample was normalized to a concentration of 1 ng µl^−1^. A 10 ng sample of DNA was used for library preparation. After tagmentation, DNA was amplified using the facility’s custom designed i7 and i5 barcodes, with 12 cycles of PCR. Due to the number of samples, the quality control for the samples was done by sequencing a pool of samples using the MiSeq V2 Nano kit – 300 cycles. Briefly, after library amplification, a 3 µl sample of each library was added to a library pool. The pool was then cleaned up using SPRIselect beads (Beckman Coulter, USA) following the Hackflex protocol. The pool was sequenced using the MiSeq V2 nano kit (Illumina, USA). Based on the sequencing data generated, the read count for each sample was used to identify the failed libraries (i.e. libraries with <100 reads), and normalized to ensure equal representation in the final pool. The final pool was sequenced on one lane of an Illumina Novaseq S4 flow cell, 2×150 bp at Novogene (Singapore).

### Genome assembly and gene screening

A modular analysis pipeline known as pipelord2, implemented with the Snakemake workflow management system, was used to perform primary bioinformatic analysis [35]. This pipeline is freely available to download from https://github.com/maxlcummins/pipelord2_0. Default settings were used unless otherwise stated. Firstly, fastp (0.20.1) was used to confirm read quality and filter poor quality reads. Kraken2 was applied to the filtered sequence reads to confirm that all genomes were *E. coli *. Draft genomes were then assembled with Shovill 1.0.4 (https://github.com/tseemann/shovill), with default settings and assembly-stats run to confirm the quality of the assemblies (https://github.com/sanger-pathogens/assembly-stats). Assemblies with >800 contigs or total length <4.5 or >6.5 Mbp were excluded. MLST 2.19.0 (https://github.com/tseemann/mlst) was used to confirm that all genomes belonged to ST372 [36]. Prokka (1.14.6) was used with default settings to annotate assembled genomes [37]. ABRicate 1.0.1 (https://github.com/tseemann/abricate) was used to screen draft genomes for genes from several publicly available and custom in-house databases. The public databases used were CARD, VFDB, PlasmidFinder, SerotypeFinder and ISFinder [38–42]. The custom database included the set of genes used to infer ColV plasmid carriage and additional virulence genes. This is available at https://github.com/maxlcummins/custom_DBs. ABRicate was also used to align assemblies to the reference pUTI89 plasmid from the *E. coli* strain UTI89, sourced from GenBank (gb | NC_007941) as well as the genomic islands that we identified. The pMLST tool available at https://bitbucket.org/genomicepidemiology/cge-tools-docker/src/master/ was used to perform pMLST [38]. AMR-associated SNPs were identified with PointFinder [37]. Finally, gene screening results are summarized by abricateR (https://github.com/maxlcummins/abricateR), with a gene being considered present at 95 % length and 90 % nucleotide identity.

### Inference of plasmid and genomic island presence in draft assemblies

The presence of a ColV type plasmid was inferred using criteria previously described by Liu *et al*. [43]. The presence of a pUTI89-like plasmid was inferred if a given assembly mapped to ≥90 % of the pUTI89 sequence at ≥90 % identity or if the isolate was determined by pMLST to carry the F29:A-:B10 RST combination, which is characteristic of pUTI89-like plasmids. Briefly, ABRicate was used to align all sequences to pUTI89 and genomic islands and plasmidmapR (https://github.com/maxlcummins/plasmidmapR) was used to bin hits into 100 bp windows that could then be visualized as heatmaps. Heatmaps were then aligned to scaled schematic representations of plasmids or genomic island-associated loci.

### Phylogenetic and SNP distance analyses

The core and pan-genomes of the annotated *E. coli* ST372 genomes and an ST127 outgroup strain SRR5336297 were determined with Roary 3.13.0 with default settings and paralogue splitting on [44]. The resulting core gene alignment of 3 160 664 bp was then used as the basis for subsequent analyses. IQTree 2.0.3 was used to infer a maximum-likelihood phylogenetic tree from the core gene alignment using the GTR+F+R substitution model and 1000 bootstrap replicates [45]. FigTree 1.4.4 (https://github.com/rambaut/figtree) was used to root the tree on the outgroup sequence, and subsequently remove it for tree visualization. snp-sites 2.5.1 was run on the core gene alignment to identify core variable SNP sites, resulting in a core SNP alignment of 22 504 bp [46]. Pairwise SNPs were extracted from the core SNP alignment with snp-dists 0.6.3 (https://github.com/tseemann/snp-dists). Fastbaps was used with a ‘baps’ prior to define clusters of isolates based on the core gene alignment and maximum-likelihood tree [47].

### Pan-genome-wide association studies (panGWAS)

Scoary 1.6.16 was used to determine associations between fastbaps cluster membership and genes in the ST372 pan-genome [48]. A Benjamini–Hochberg-adjusted *P*-value cutoff of 1E-20 was used to determine significant associations. The *P*-value was selected with two considerations in mind, (a) to be very low in order to cut out the amount of noise Scoary generates via multiple comparisons despite the Benjamini–Hochberg adjustment, and (b) to provide a manageable number of gene candidates with putative functions for further validation. Biological process terms associated with the identified genes were derived from UniProt entries for each gene.

### Identification and characterization of genomic islands

Genomes that carried genes representative of genotypes identified in the GWAS analysis were selected. GenBank files were uploaded to IslandViewer 4 (https://www.pathogenomics.sfu.ca/islandviewer/) and aligned to *E. coli* ST127 strain ECONIH2 (gb|CP014667.1) as a reference sequence [49]. Annotation files with predicted genomic islands were downloaded and annotated in SnapGene Viewer (version 5.0.7, GSL Biotech LLC).

### Data analysis and visualization

A custom R script was written in RStudio 1.4.1106 with R 4.1.3 to perform secondary analysis on the data generated by pipelord2 and the phylogenetic methods described above. The script also produces the publication figures, excepting some manual editing that was necessary for ease of viewing and interpretation. The genomic island sequences in Fig. S2-5 were visualized with SnapGene Viewer (version 5.0.7, GSL Biotech LLC).

### Data availability

The data analysis and visualization script is available at https://github.com/CJREID/ST372 and can be used to reproduce all data analysis. R package versions used therein are available within the README.md document in the code repository.

Melbourne Veterinary Collection (MVC) genomes were deposited in GenBank and the Sequence Read Archive (SRA) under BioProject PRJNA678027. Additional ST372 genome sequences from collaborators and gull sequences were deposited under BioProject PRJNA827950. Individual SRA, BioSample and BioProject accession numbers for all sequences used in the study can be found in Table S1.

## Results

### Study collection

**Fig. 1.**
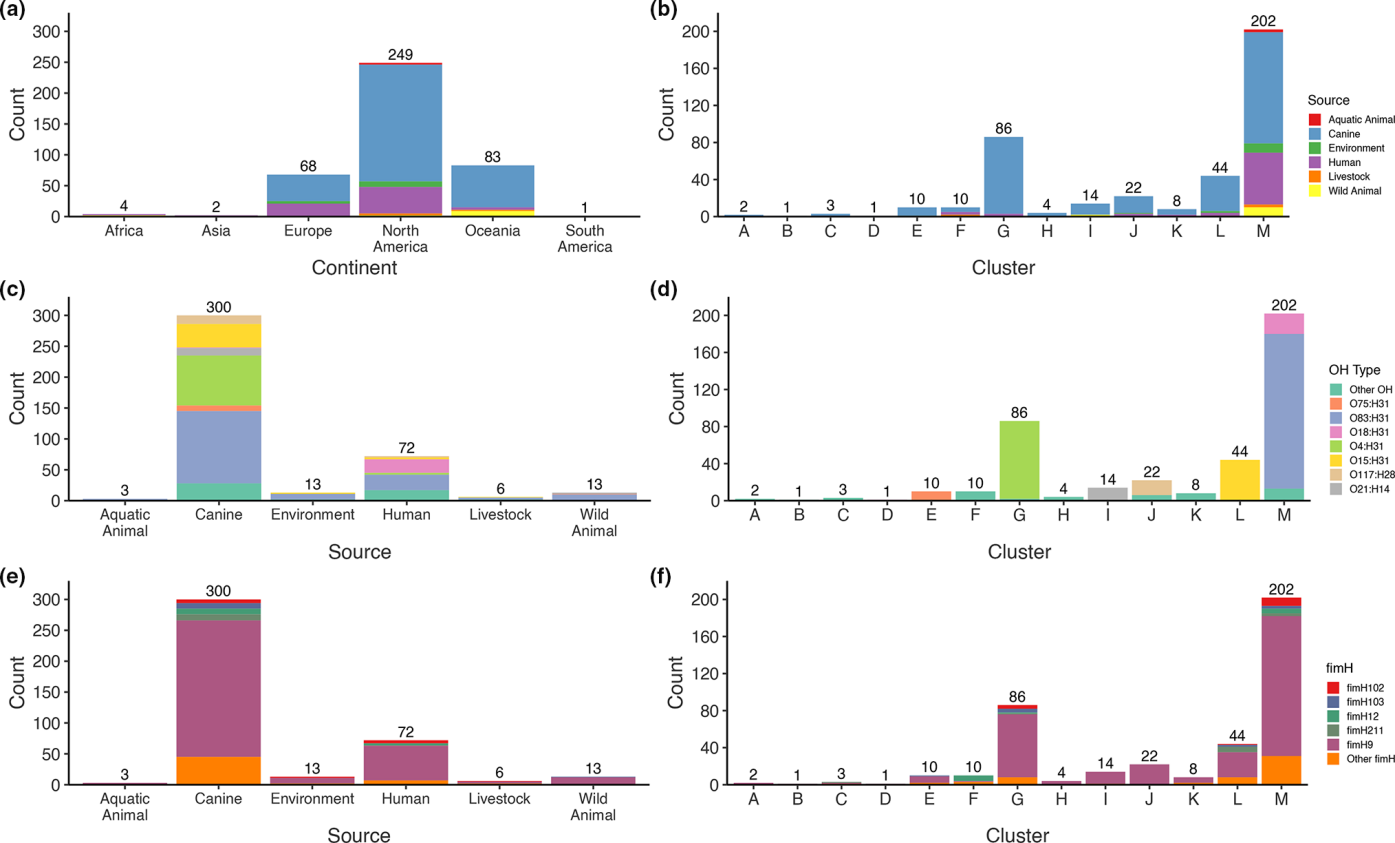
Genomic epidemiological features of the ST372 genome collection. (**a**) Distribution of continents stratified by source, (**b**) distribution of phylogenetic clusters (see Fig. 2) stratified by source, (**c**) distribution of sources stratified by O:H type, (**d**) distribution of phylogenetic clusters stratified by O:H type, (**e**) distribution of sources stratified by *fimH* allele and (f) distribution of phylogenetic clusters stratified by *fimH* allele. Total number of observations for each variable are displayed above each bar. See Fig. S1 for source-stratified graphs of OH and *fimH* data.

The study collection comprised 407 *E. coli* ST372 isolated between 1980 and 2020, and included both publicly available (*n*=285) and newly generated genome sequences (*n*=122). Sequences of canine origin dominated (300, 73.7 %), but human (72, 17.7 %), wild animal (mostly birds; 13, 3.2 %) and environmental (natural and wastewater; 13, 3.2 %) also featured (Fig. 1a). Most sequences originated from North America (249, 61.2 %), Oceania (83, 20.4 %) and Europe (68, 16.7 %) with a total of 19 countries represented (Fig. 1a, Table S1).

### Population structure and genomic epidemiology

**Fig. 2.**
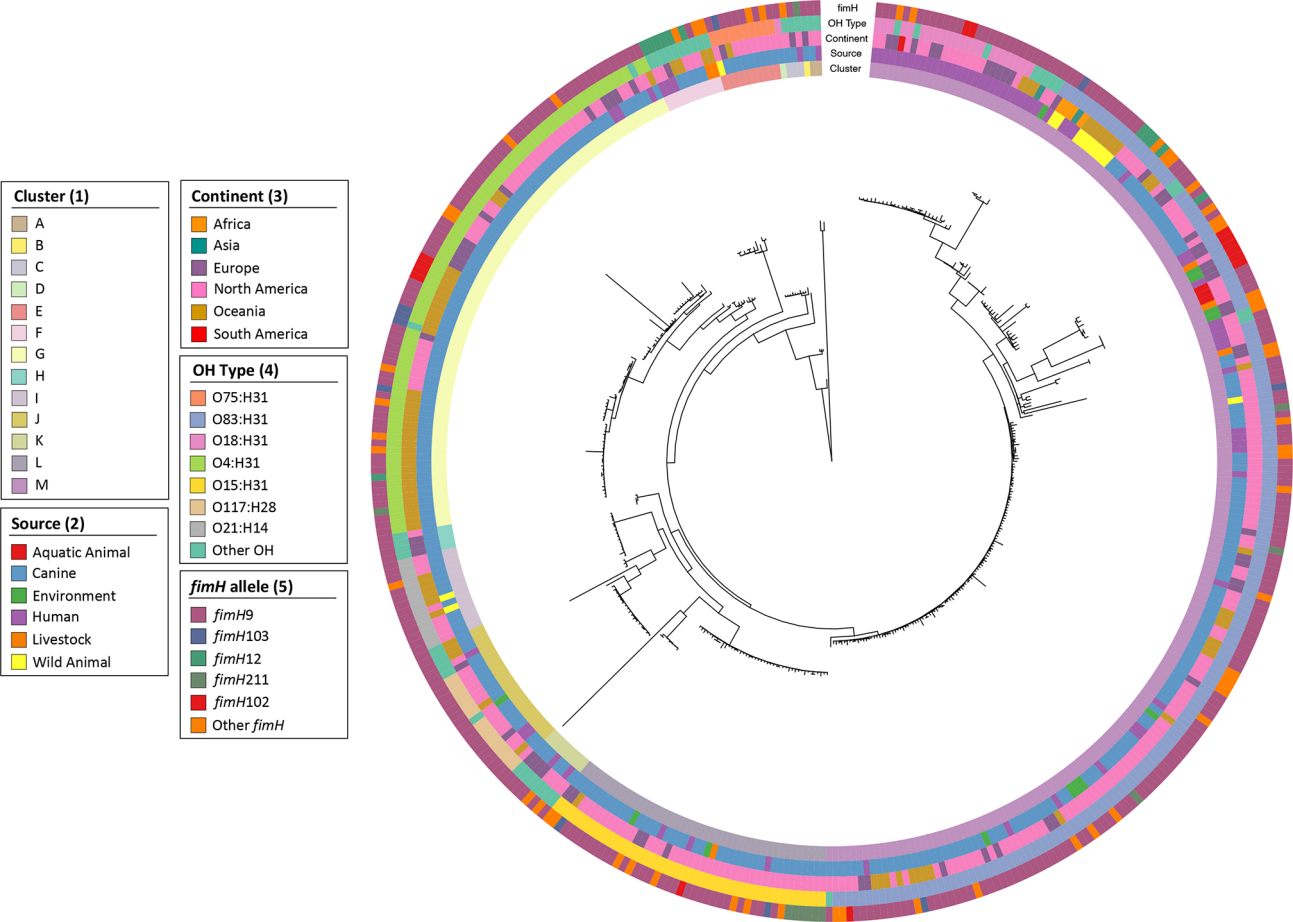
Phylogeny of ST372. A maximum-likelihood core gene phylogeny based on alignment of 3493 genes identified in all ST372 sequences. Metadata displayed from inner to outermost rings display fastbaps cluster, source, continent, O:H type and *fimH* allele.

The maximum-likelihood core genome phylogeny was inferred from a 3 160 664 bp alignment of 3493 genes identified in all ST372 sequences. The resulting tree was grouped by fastbaps into 13 clusters, which were designated letters from A–M. Cluster M was the largest (202, 49.6 %) comprising mostly canine source sequences (120/202, 59.4 %) as well as most of the human sequences in the collection (56/72, i.e. 77.8 % of human total, and 56/202, i.e. 27.7 % of cluster M) (Fig. 1b). Smaller yet sizeable clusters included G (86, 21.1 %), L (44, 10.8 %) and J (22, 5.41 %); all of which were canine-dominated but also featured human sequences. In cluster M, 29 human sequences and 1 environmental sequence from multiple continents formed a divergent clade from the remainder of the cluster. This human-dominated clade mostly carried O18:H31 O:H type in contrast to the remainder of cluster M, which was predominantly O83:H31 (Fig. 2). Apart from this split within cluster M, different O:H types generally corresponded tightly with cluster but not source (Fig. 1c, d). For example, clusters G, L and J primarily carried O4:H31, O15:H31 and O117:H28 O:H types, respectively; all of which were shared between canine- and human-source sequences. Unlike O:H types, *fimH* alleles did not show correspondence with cluster. *fimH9* was the major *fimH* allele (304, 74.7 %) and was identified in all sources and clusters. Overall, the general phylogenetic and epidemiological analyses indicated that the ST372 population structure features multiple O:H type-delineated clusters where human source sequences intermingle with the dominant canine source sequences. The dominant cluster M generally follows this trend but also contains a human-dominated clade.

### Core genomic linkage between canine and non-canine origin sequences

**Fig. 3.**
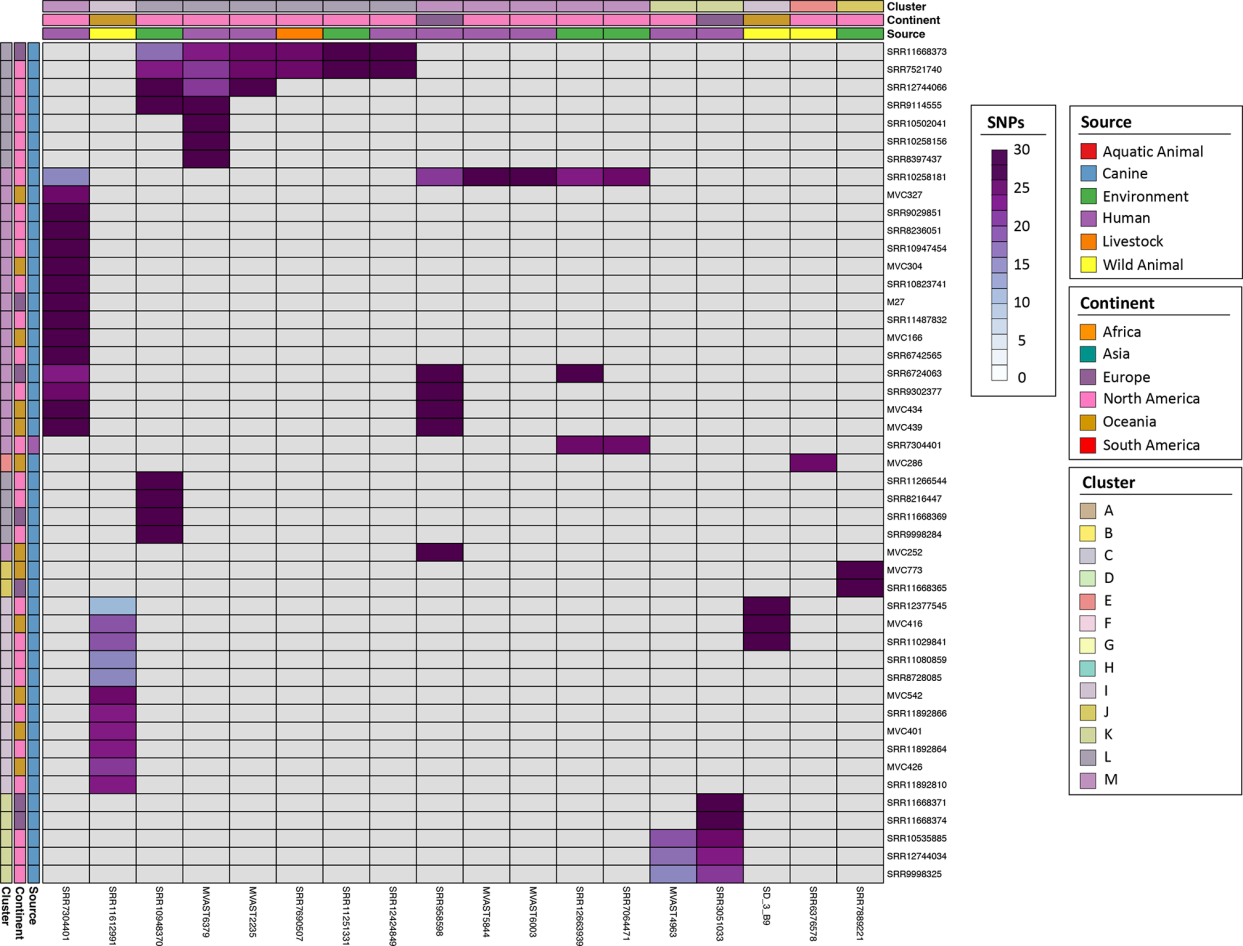
Low pairwise SNP distance heatmap between ST372 isolated from canine and non-identical sources. Metadata are represented for each sequence in coloured bars for each row and column. Each cell in the heatmap represents a pairwise SNP comparison between two sequences represented by white to purple gradient. Grey squares represent distances of >30 SNPs.

To obtain greater resolution of the potential overlap between canine and human or non-canine sourced sequences, we generated a pairwise SNP matrix of conserved variable sites extracted from the core gene alignment (22 504 total SNPs) and filtered pairs of strains that differed by 30 SNPs or less. Across the ~3.16 Mbp alignment this amounts to a core genomic divergence of ≤0.000949 % over 3493 genes. This analysis identified 77 unique pairs of closely related sequences from non-identical sources (Fig. 3). Thirty-five sequence pairs were from different continents. Source pairs were mostly canine : human (43/77) found in clusters K [8], L [12] and M [23], followed by canine : wild animal (15/77) mostly in cluster I [14] and canine : environment (15/77; all water-associated) in clusters L [10], M [3] and J [2]. Overall, this indicates the presence of intercontinental transmission pathways trafficking closely related ST372 between dogs, human and non-human sources.

### Cluster-associated accessory genome features

**Fig. 4.**
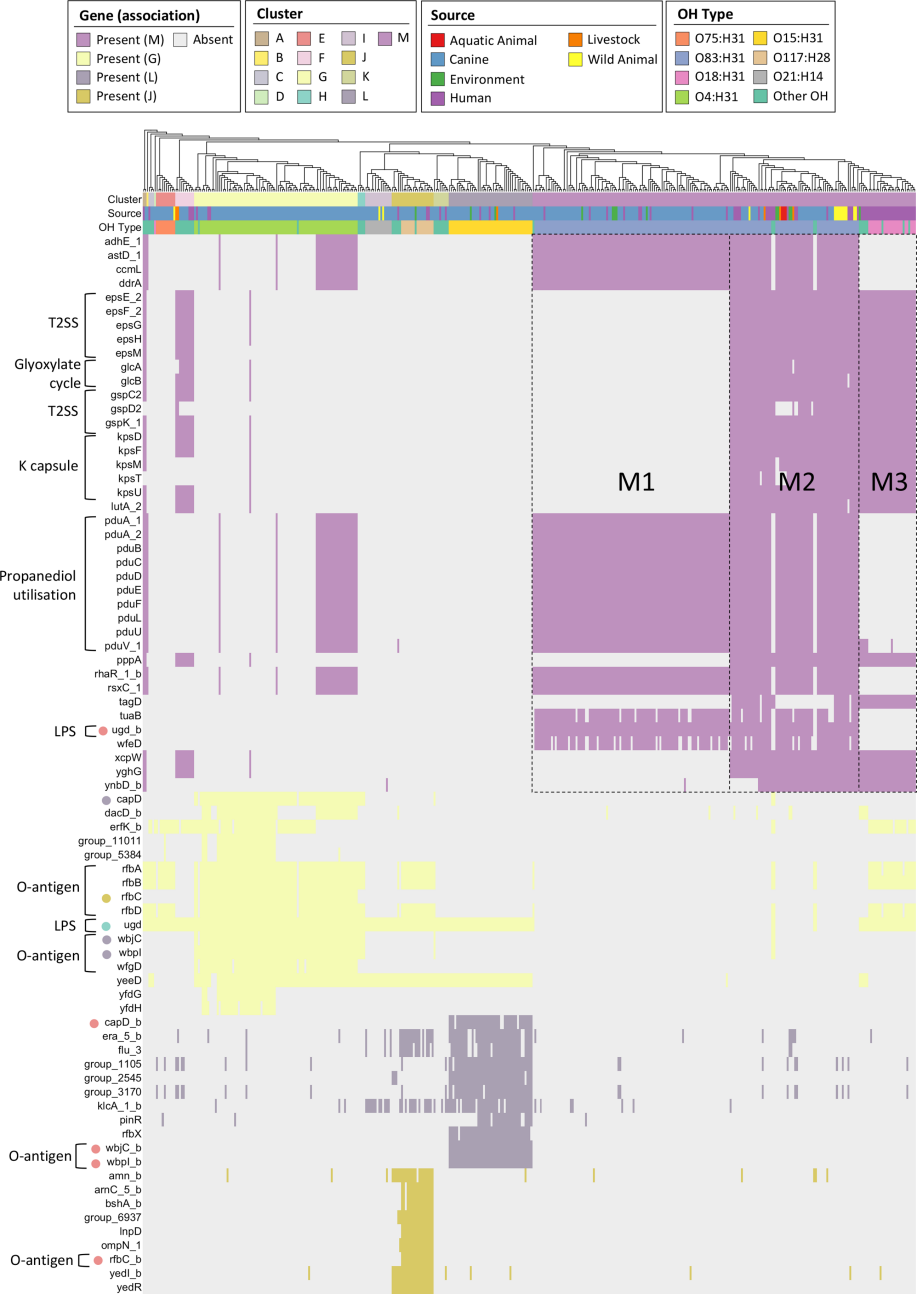
Map of cluster-associated genes aligned to the core gene phylogeny. Columns display metadata (cluster, source, O:H type) followed by genes found to be over-represented in clusters M, G, L and J. Gene presence/absence is indicated with the same colour as the associated cluster. Coloured dots next to gene names indicate where an alternative allele of the same gene is identified in another cluster. Bracketed labels indicate gene functions for select genes/operons.

A pan-GWAS analysis was utilized to identify genes with putative functions associated with the previously defined phylogenetic clusters. We identified 76 genes that were over-represented in four clusters (Fig. 4, Table S2). The largest cluster M contained 40 such genes, whilst clusters G [16], L [11] and J [9] also displayed gene associations. The most notable shared function of genes over-represented in clusters G, L and J was O-antigen biosynthesis (e.g. *rfbABCD*, *wfgD*, *wbpI*, *wbjC*), reflective of the previously noted correspondence between cluster and O:H type. Otherwise, genes indicative of selection for specific metabolic or pathogenic traits in these smaller clusters were not obvious. In contrast, cluster M displayed several intriguing genetic features, including propanediol metabolism operon (*pdu*), a type II secretion system (T2SS; *eps/gsp*) and K capsule (*kpsMT*). The *pdu* operon as characterized in *Salmonella* facilitates microcompartment-mediated metabolism of 1,2 propanediol (also known as propylene glycol) – a common additive to commercial dog food. Mapping the presence of these genes back to the phylogeny revealed that these genes were not uniformly distributed within cluster M. Instead, there were three apparent accessory genotypes, which we designated M1, M2 and M3. The M1 genotype comprised uniform carriage of the *pdu* operon genes, *adhE*, *astD*, *ccmL*, *ddrA*, *rhaR* and *rsxC,* with variable yet consistent presence of *tuaB*, *ugd* and *wfeD*. M2 comprised the M1 genes in addition to variable yet consistent carriage of *eps* and *gsp* T2SS genes, *kps* operon, *glcAB*, *lutA*, *pppA*, *xcpW*, *yghG* and *ynbD*. The M3 genotype was essentially the M2 genotype minus the M1 genotype and therefore comprised *eps* and *gsp* genes, *kps* operon, *glcAB*, *pppA*, *tagD*, *xcpW*, *yghG* and *ynbD*. Similar gene carriage patterns to the M subgroups were also observed in clusters G, F, A and B, for example *pdu* genes were not found to be associated with cluster G but were present in a subset of cluster G sequences.

### Genomic context of cluster-associated genes

**Fig. 5.**
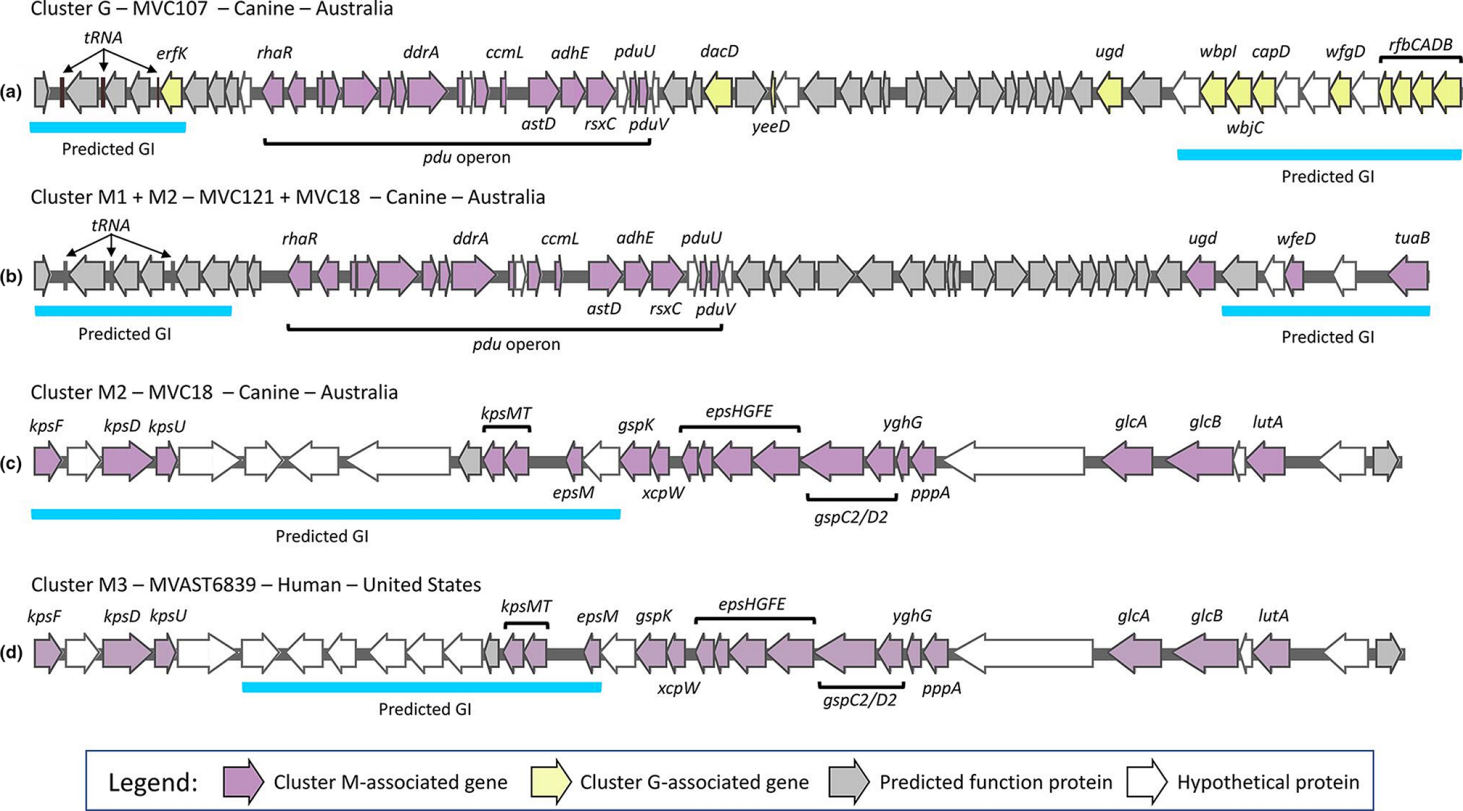
Schematic representations of representative gene loci containing cluster-associated genes (see Fig. 4) and predicted genomic islands. (**a**) Cluster G-associated genes identified in proximity to *pdu* operon (cluster M-associated) represented by cluster G sequence MVC107, (**b**) cluster M-associated genes (*pdu* operon), as seen in M1 and M2 genotypes from Fig. 4. (represented by sequences MVC121 and MVC18, respectively), (**c**) cluster M-associated associated genes (*kps* operon, *eps*, *gsp*) represented by M2 genotype sequence MVC18 and (d) cluster M-associated genes (*kps* operon, *eps*, *gsp*) represented by M3 genotype sequence MVAST6839.

The above results indicated that multiple genes of interest in ST372, particularly the *pdu* operon, were gained and lost together, suggestive of horizontal gene transfer. We therefore selected representative sequences of cluster G (MVC107 – canine), M1 (MVC121 – canine), M2 (MVC18 – canine) and M3 (MVAST6839 – human) genotypes and examined the annotated contigs for gene co-carriage as well as evidence of genomic islands (Fig. 5). We also aligned the rest of the genome collection to these representative sequences to infer their presence in other isolates. This analysis confirmed that almost all the genes suspected to be linked could be found on the same assembly scaffold. In MVC107 (cluster G), the *pdu* operon was identified between two predicted genomic island regions. The upstream island contained multiple tRNA genes, whilst the downstream island contained cluster G-associated O-antigen biosynthesis genes (Fig. 5a). The genomic regions containing the *pdu* operon in MVC121 (M1 genotype) and MVC18 (M2 genotype) were identical and found between two predicted genomic island regions. The upstream island was broadly similar to that in MVC107 containing three tRNA genes, but the downstream island gene was different and included cluster M-associated genes *wfeD* and *tuaB*. The cluster M-associated *ugd* gene allele, in contrast to the cluster G-associated *ugd* allele, was also present. Alignment of cluster G and cluster M1/M2 *pdu* operon contigs to the remainder of the collection supported their presence in sequences belonging to these groups (Figs S2 and S3). Sequences in clusters A and B that evidently carried the *pdu* operon did not map completely to either the G or M1/M2 arrangements, indicating an alternative genomic context for *pdu* in those genomes. The *pdu* operon identified in MVC121 was structurally similar to that described in *Salmonella enterica* serovar Typhimurium strain LT2 (gb|AF026270.2), mapping to 94 % of the sequence found in LT2 but displaying an average nucleotide identity of just 77.49 % (Table S3). These data suggest that the operon found in *E. coli* is distantly related to that found in *Salmonella,* yet likely serves a similar function. In sum, these results indicate that the *pdu* operon has been acquired in ST372 from divergent genomic origins on multiple occasions in association with genomic islands. The mechanism of their acquisition cannot be reliably linked to genomic island mobility by our data alone, and may equally have occurred due to homologous recombination or the activity of other mobile genetic elements.

Analysis of genes found in the M2 and M3 genotypes revealed at least two genomic contexts for the K capsule and T2SS genes. In MVC18 (M2), all *kps* genes were predicted to belong to a genomic island containing some additional predicted ORFs, present upstream of the *eps*, *gsp*, *glcAB* and *lutA* genes (Fig. 5c). In MVAST6839 (M3), the same gene repertoire was present, but the region predicted to belong to a genomic island only contained the *kpsMT* genes and several predicted ORFs that differed from the arrangement seen in MVC18 (Fig. 5d). Mapping these contigs back to the full collection showed that the MVC18 arrangement was only conserved within a subset of M2 sequences, whereas the MVAST6839 arrangement was present in both M2 and M3, indicating that it has been acquired more than once in ST372 (Figs S4 and S5). As above, these results indicate multiple gene acquisition events, whereby K capsule and T2SS genes have been acquired from differing origins.

## Discussion

Here we assembled a collection of all available *E. coli* ST372 genomes to investigate their genomic epidemiological features. As suggested by previous studies, we confirm that ST372 is primarily a canine-associated lineage and has a population structure with multiple clusters broadly identifiable by O:H type. We found evidence of very closely related sequences from dogs and humans, indicative of interspecies transmission, and an apparently human-specific lineage emerging from the largest cluster, cluster M. Furthermore, we found that multiple gene gain and loss events associated with predicted genomic islands differentiate subgroups within cluster M.

### Genomic epidemiological features of *E. coli* ST372

Despite our efforts to generate a genome collection from diverse sources, the collection was dominated (73.7 %) by isolates from dogs. Our findings and those of others strongly support ST372 as the major *E. coli* sequence type colonizing and causing infections in dogs in Europe, the USA and Australia [16, 17, 20]. We acknowledge that sequences from Asia and Africa were rare and although we suspect that the distribution of ST372 is global, we cannot exclude the possibility that dogs in these regions might be predominantly colonized by other *E. coli* STs reflective of unknown region-specific influences.

The population structure, as defined by fastBAPS clusters, mostly corresponded with single O:H types. The largest cluster (cluster M), however, which mainly comprised O83:H31 strains (167/202, 82.7 %), also included O18:H31 (22/202, 10.9 %) and O45:H31 (5/202, 2.5 %). The O18:H31 and O45:H31 sequences were from multiple continents, exclusively of human source, and each split into their own clades at the most divergent end of the phylogeny. This suggests serotype-mediated host adaptation, though other genomic features are likely to have been involved, as discussed later. Flament-Simon *et al*. previously noted an association between O18:H31 and O45:H31 O:H types and human source in ST372 [16]. Correlations between host, O:H type and specific mobile genetic elements have previously been noted in Shiga-toxigenic *E. coli* from sheep and cattle, *Salmonella* and *Klebsiella pneumoniae* [50–53]. Taken collectively, these studies indicate that whilst host selection for serotype is an obvious explanation for host–O:H type correlations, host selection of mobile genetic elements, the presence of which are mediated by specific serotypes, is also likely to be a contributing factor. Data from our panGWAS analysis, discussed later, support this idea. Overall, our results indicate that whilst most clusters of ST372 are canine-adapted, some are human-adapted and belong to sub-lineages with different O:H types that have emerged from the canine-adapted genetic background. Whether this phenomenon should be included within the standard conception of zoonosis warrants further consideration.

### Evidence of ST372 zoonosis

In support of the zoonotic potential of *E. coli* ST372, we identified 43 canine–human sequence pairs whose members differed by fewer than 30 core genome SNPs, most of which (23/43) fell within the canine-dominated portion of cluster M. For epidemiologically unrelated isolates, this represents an extremely high level of relatedness; a difference of ≤0.00095 % of the core genome. For perspective, previous studies on hospital-based outbreaks of *E. coli* have identified transmission with SNP thresholds ranging from 17 to 23 SNPs [54]. It should be noted that the ability to use a true ‘reference’ isolate in an outbreak scenario results in SNP counts calculated over a near complete alignment of two genomes as opposed to core genes only, as in our study. This results in a much lower percentage difference than we observe. However, given the significant differences between spatiotemporally restricted human-to-human transmission in a hospital and potentially global scale transmission over expanses of time between different species, we believe our approach is suitable for inferring the occurrence of interspecies transfer in global-scale collections. Further supporting this contention, similar results were observed previously between epidemiologically unrelated sequences within other important STs suspected to have zoonotic potential, including ST58, ST95 and ST127 [4, 6, 21].

We note that the present collection is heavily weighted towards clinical isolates from both dogs and humans, despite the fact that the gastrointestinal tract is the major reservoir of such isolates, wherein ST372 may reside for long periods before causing infection, or without ever causing infection. Given the fact that we identified close linkage between infectious isolates separated geographically and by unknown periods of gastrointestinal colonization time in different hosts, we suspect that contemporaneous, paired faecal isolates of ST372 from dogs and humans within the same household would exhibit outbreak-level similarity. As such, the level of core genomic similarity observed within this unlinked collection could be considered a globalized reflection of highly related strains, shared via repetitive within-household transmission of ST372 between humans and their pet dogs via shared living space, physical intimacy and shared diets. Whether diet is a point source of ST372 for dogs and humans remains unknown but may be important. Foodborne sources of ST372 that might drive carriage by dogs and humans are worthy of further investigation, though we note a low level of livestock sequences in this collection (*n*=6). This result may be due to generally low levels of livestock- or meat-source *E. coli* genomes that are publicly available or because ST372 is not a frequent colonizer of food animals and their meat products. To the best of our knowledge, household transmission of ST372 has yet to be reported, although numerous studies demonstrate or infer transmission of *E. coli* between dogs and their owners, supporting direct transmission as a likely reason for the presence of ST372 in both dogs and humans [22, 25, 55, 56]. The lack of ST372 in these studies might be due to the widespread practice of selection for isolates that display resistance to high-importance antimicrobials, traits that are uncommon in ST372 *E. coli* [16]. Such practices obscure a wealth of selection pressures driving the evolution and emergence of gut-colonizing opportunistic pathogens such as *E. coli *.

### Two types of zoonosis?

Whilst the short-term direct transfer of a pathogenic organism from a primary to a secondary host is a classical conception of zoonosis, the divergent evolution of a pathogen adapted to the secondary host from the primary host genotype over a longer time span should equally be considered a form of zoonosis. The latter scenario cannot be detected via traditional epidemiological approaches, but genomic epidemiology, which pairs core gene phylogeny, accessory gene content and host metadata, allows such inferences to be made. We believe that our data indicate that both zoonotic scenarios have occurred within the history of *E. coli* ST372, as evidenced by (a) the low SNP counts between sequences in the same evolutionary clusters and (b) the emergence of a human-restricted lineage with distinctive genomic traits. It is intuitive that the greater the frequency of classical or ‘direct’ zoonosis of a pathogen from a primary host to a secondary host (e.g. dogs to humans), the greater the likelihood that a lineage adapted to the secondary host might emerge. However, a significant amount of additional data describing actual or inferred rates of transfer would be required to test this hypothesis. The case of human-adapted ST131 *E. coli* spilling into dogs in conjunction with the emergence of dog-adapted lineages of ST131 is a strikingly similar scenario to what we propose in ST372, albeit in the opposite direction [57].

### Genomic island-associated genes under selection in canine and human ST372 strains

What underlies the association of ST372 with dogs, and the development of human-associated ST372 lineages? The large pan-genome of *E. coli* includes an array of accessory genes that confer diverse adaptive capabilities to strains that possess them. As such, accessory genomes may provide a wealth of information about the evolution and adaptation of STs and their sub-lineages.

In the ST372 pan-genome we identified 40 accessory genes that were associated with the largest cluster, cluster M. Among these were genes of the *pdu* operon. This operon was originally described for its involvement in anaerobic microcompartment-mediated metabolism of glycerol and 1,2 propanediol in *S. enterica* serovar Typhimurium, although it is found in a wide range of enteric and soil-dwelling bacteria [58–62]. In *S. enterica,* the operon has roles in colonization of the gastrointestinal lumen and in pathogenicity [63–65]. In the foodborne pathogen *Listeria monocytogenes* the *pdu* operon is similarly noted for its role in gut colonization, virulence, and persistence on food [66]. Functional expression of the operon was also retained upon cloning from *S. enterica* into a variety of Gram-negative species, including *E. coli* [67]. Given that glycerol and 1,2 propanediol are common additives in semi-moist commercially available dog food, it seems less than coincidental that the major group of *E. coli* colonizing and causing infections in dogs carries genes that facilitate their metabolism. Could commercial dog food have contributed to the evolution of a canine pathogen? A similar hypothesis of diet-driven selection was presented as an explanation for the emergence of an IncHI1 plasmid carrying specific metabolic genes, circulating in ST1250 *E. coli* from horses in Europe [68]. The fact that the operon was not ubiquitous in canine-source ST372 indicates that other traits have contributed to the prominence of ST372 in dogs. Nonetheless, the high frequency of the *pdu* operon within the largest cluster, cluster M, and its occurrence in several other clusters in association with different predicted genomic islands show that it has been selected multiple times, plausibly by dietary factors. Further work is required to conclusively assess these hypotheses.

A proportion of sequences in cluster M contained genomic island-associated K capsule genes in two arrangements. The K antigen locus is phage-mobilized and has well-described functionality in human pathogenesis and possible roles in gut colonization, likely explaining the apparent selection of these genes in human-restricted clades of ST372 and in branches of the phylogeny immediately basal to these clades [69–71]. The pattern of *pdu* and *kps* carriage we observed in cluster M support (a) the initial acquisition of the *pdu* island in cluster M, mostly in association with dogs, defining the M1 genotype; (b) at least two acquisitions of the distinct K antigen islands seen in the M2 genotype, in conjunction with emergence in additional non-human hosts; and (c) eventual loss of the *pdu* operon in the M3 genotype, contemporaneous with a switch in O:H type favouring human host-specificity.

As was suggested earlier, O:H type might have a role to play in the selection of accessory genes in conjunction with the host. The human-restricted lineages of cluster M carried O:H types O45:H31 and O18:H31, in contrast to the remainder of cluster M, which carried O83:H31. Transition to human host in conjunction with a loss of O83:H31 might explain the loss of the *pdu* operon genes as a result of lack of selective pressure in humans for *pdu* and exclusion of the genomic island by the alternative serotypes. This supports a paradigm whereby mobile genetic elements carrying functionally beneficial gene combinations are selected via interaction with bacterial serotype and factors related to the animal host.

## Conclusions

Our results indicate that dogs are the primary reservoir of ST372 and major contributors to the evolution of this lineage. The data support transfer of pathogenic isolates between dogs and humans and emergence of a human-adapted lineage from the canine-dominated population. We highlight two horizontally transferred genomic islands that are apparently associated with evolution and selection in dog and human hosts. Whilst these provide partial explanations for the success of *E. coli* ST372, further genomic and phenotypic studies are required to fully understand its emergence and evolution.

## Supplementary Data

Supplementary material 1

Supplementary material 2
