# Corrigendum: Identification of genes influencing the evolution of Escherichia coli ST372 in dogs and humans

**DOI:** 10.1099/mgen.0.001286

**Published:** 2024-08-21

**Authors:** Paarthiphan Elankumaran, Glenn F. Browning, Marc S. Marenda, Amanda Kidsley, Marwan Osman, Marisa Haenni, James R. Johnson, Darren J. Trott, Cameron J. Reid, Steven P. Djordjevic

**Affiliations:** 1 Australian Institute for Microbiology and Infection, School of Life Sciences, Faculty of Science, University of Technology Sydney, Ultimo, NSW, Australia; 2 Asia- Pacific Centre for Animal Health, Department of Veterinary Biosciences, Melbourne Veterinary School, Faculty of Veterinary and Agricultural Sciences, University of Melbourne, Parkville and Werribee, Victoria, Australia; 3 Australian Centre for Antimicrobial Resistance Ecology, School of Animal and Veterinary Sciences, University of Adelaide, Roseworthy, Australia; 4 Laboratoire Microbiologie Santé et Environnement, Doctoral School of Sciences and Technology, Faculty of Public Health, Lebanese University, Tripoli, Lebanon; 5 Department of Public and Ecosystem Health, College of Veterinary Medicine, Cornell University, Ithaca, NY, USA; 6 ANSES, Université de Lyon, Unité Antibiorésistance et Virulence Bactériennes, Lyon, France; 7 Minneapolis VA Medical Center, Minneapolis, MN, USA

In the published version of this article, there was a minor typographical error in the surname of the first author Paarthiphan Elankumaran. In the original article the name appears as ‘Paarthiphan Elankum**u**ran’, which has been corrected to ‘Paarthiphan Elankum**a**ran’.

The Authors apologise for any inconvenience caused.

