# Supplementary material for: Identification of genes influencing the evolution of Escherichia coli ST372 in dogs and humans
Source: Microb Genom. 2023 Feb 8;9(2):mgen000930. doi: 10.1099/mgen.0.000930 (PMC9997745; doi:10.1099/mgen.0.000930)
Supplement: Supplementary material 1 [file mgen-9-930-s001.pdf]

Supplementary Figures for: Elankumaran *et al,* Identification of genes influencing the evolution of *Escherichia coli* ST372 in dogs and humans

Figure Legends

Figure S1. Summary of OH Type and Serotype stratified by source for ST372 sequences

Figure S2. Alignment of ST372 sequences against genomic island identified in strain MVC107 and shown in Fig. 5a. Heatmap shows presence/absence of >90% nucleotide identity across 100 bp segments of the genomic island.

Figure S3. Alignment of ST372 sequences against genomic island identified in strains MVC121 and MVC18, and shown in Fig. 5b. Heatmap shows presence/absence of >90% nucleotide identity across 100 bp segments of the genomic island.

Figure S4. Alignment of ST372 sequences against genomic island identified in strain MVC18 and shown in Fig. 5c. Heatmap shows presence/absence of >90% nucleotide identity across 100 bp segments of the genomic island.

Figure S5. Alignment of ST372 sequences against genomic island identified in strain MVAST6839 and shown in Fig. 5d. Heatmap shows presence/absence of >90% nucleotide identity across 100 bp segments of the genomic island.

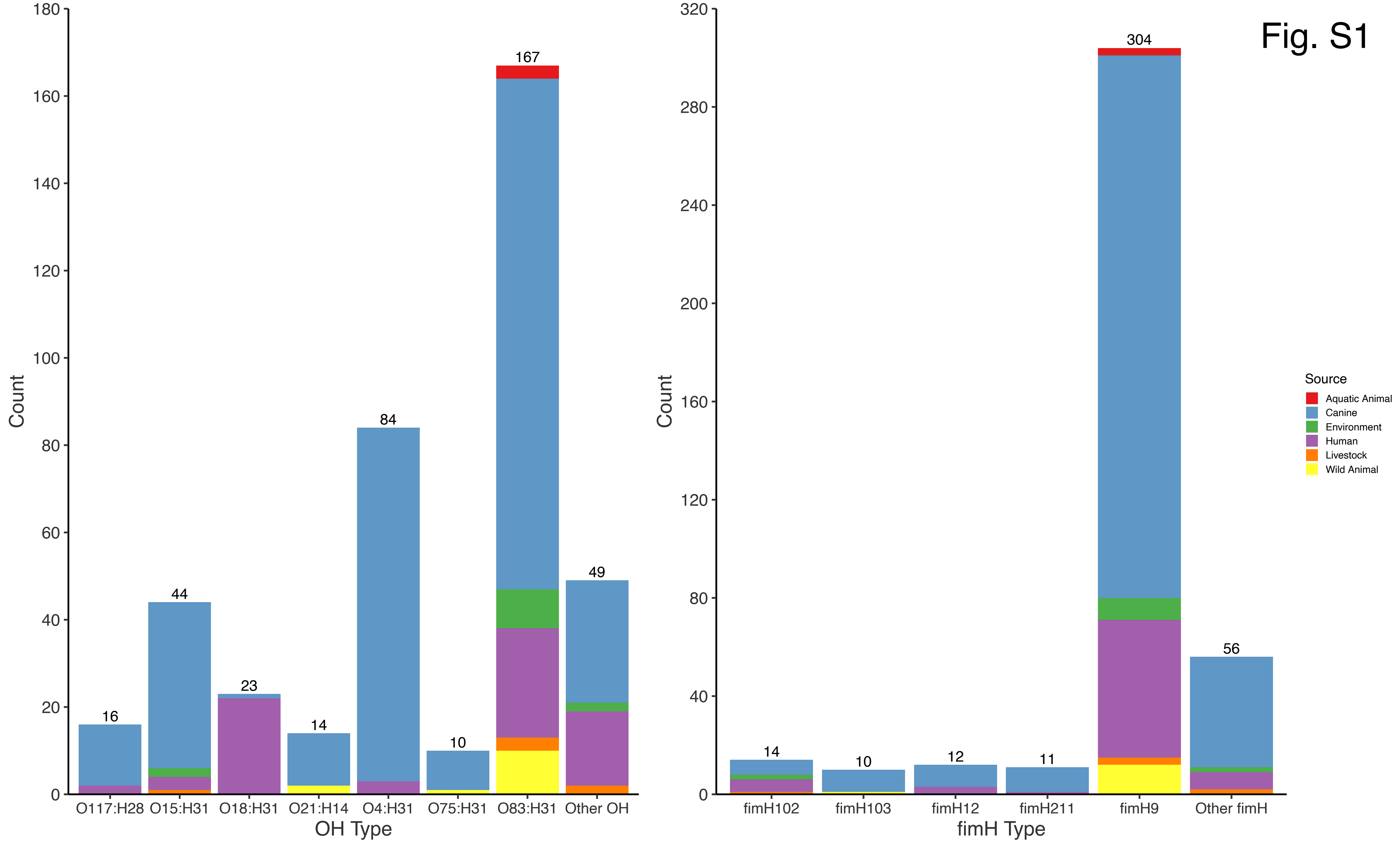
Fig. S1
Count
OH Type
O117:H28
O15:H31
O18:H31
O21:H14
O4:H31
O75:H31
O83:H31
Other OH
16
44
23
14
84
10
167
49
fimH Type
fimH102
fimH103
fimH12
fimH211
fimH9
Other fimH
14
10
12
11
304
56
Source
Aquatic Animal
Canine
Environment
Human
Livestock
Wild Animal

Fig. S2

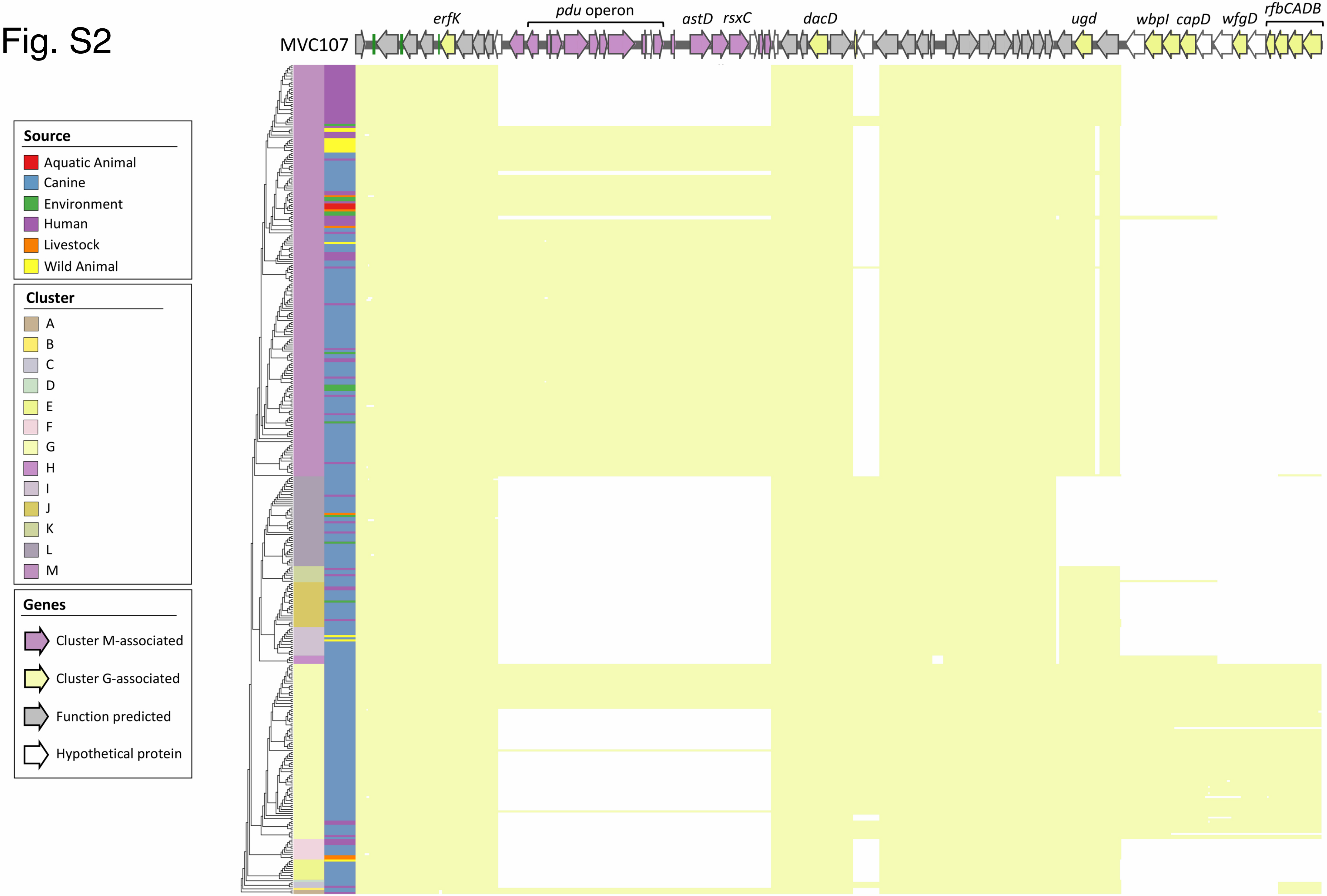
MVC107
erfK
pdu operon
astD
rsxC
dacD
ugd
wbpI
capD
wfgD
rfbCADB
Source
Aquatic Animal
Canine
Environment
Human
Livestock
Wild Animal
Cluster
A
B
C
D
E
F
G
H
I
J
K
L
M
Genes
Cluster M-associated
Cluster G-associated
Function predicted
Hypothetical protein

Fig. S3

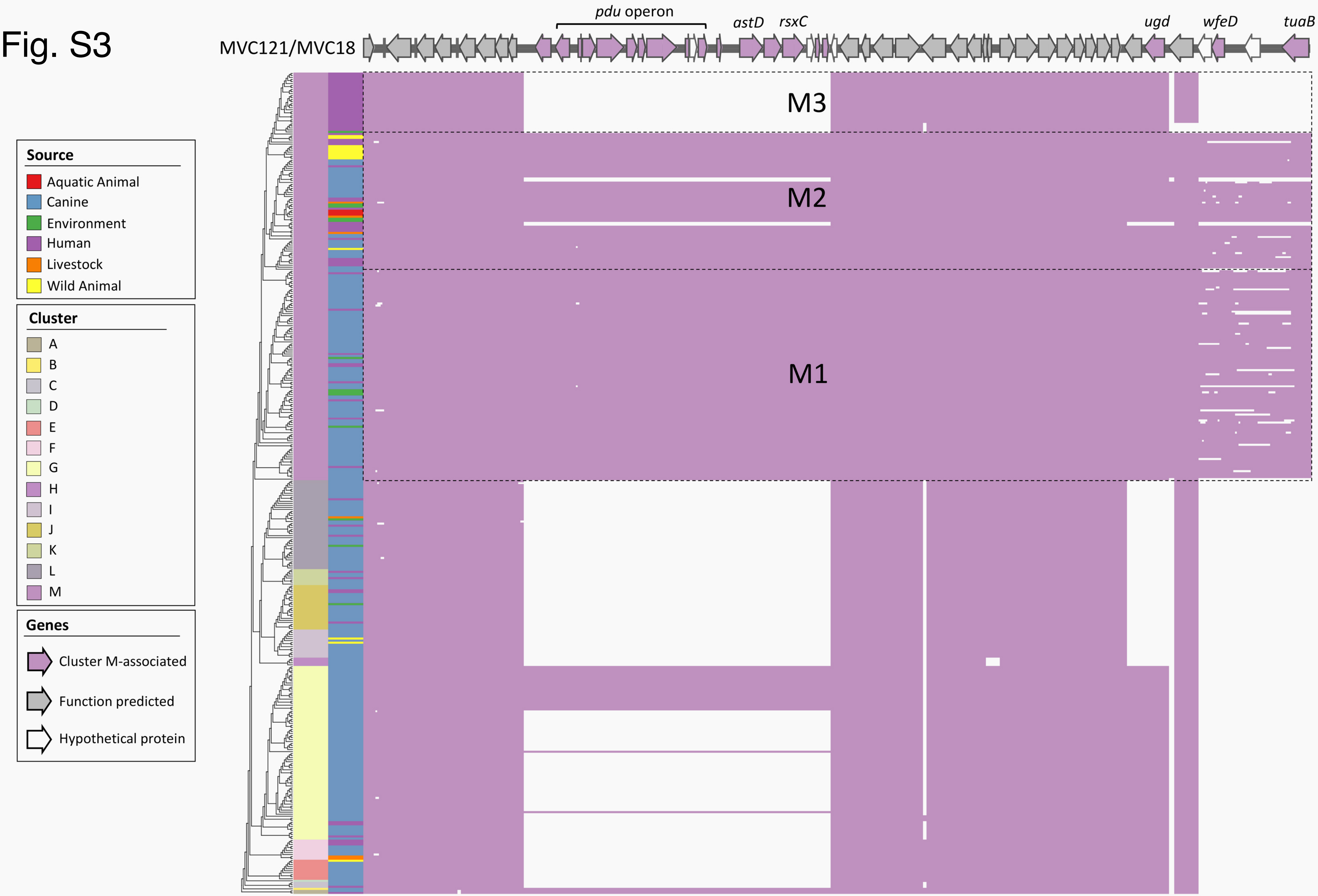

MVC121/MVC18
pdu operon
astD
rsxC
ugd
wfeD
tuaB
M3
M2
M1
Source
Aquatic Animal
Canine
Environment
Human
Livestock
Wild Animal
Cluster
A
B
C
D
E
F
G
H
I
J
K
L
M
Genes
Cluster M-associated
Function predicted
Hypothetical protein

Fig. S4

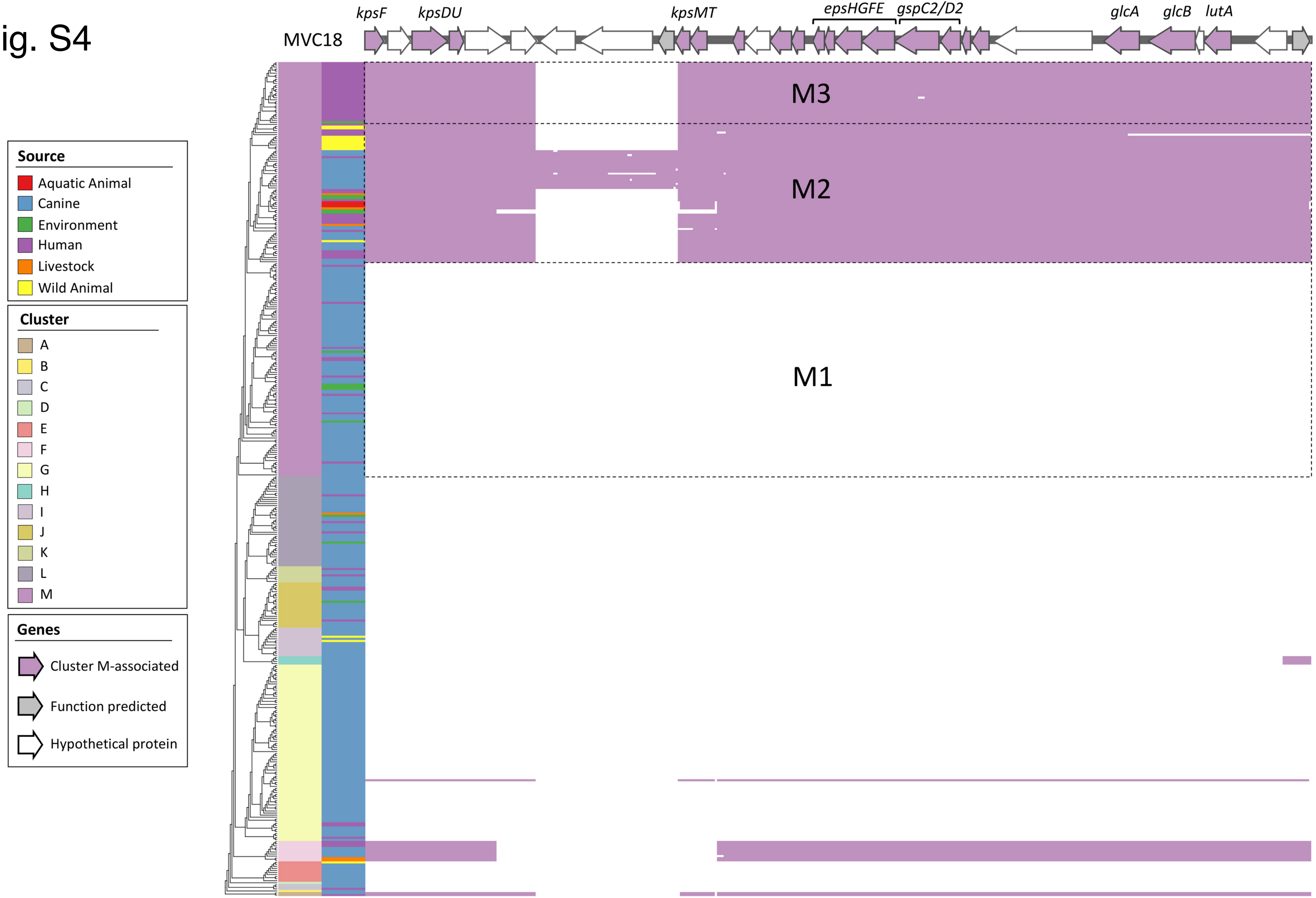
kpsF
kpsDU
kpsMT
epsHGFE
gspC2/D2
glcA
glcB
lutA
MVC18
M3
M2
M1
Source
Aquatic Animal
Canine
Environment
Human
Livestock
Wild Animal
Cluster
A
B
C
D
E
F
G
H
I
J
K
L
M
Genes
Cluster M-associated
Function predicted
Hypothetical protein

Fig. S5

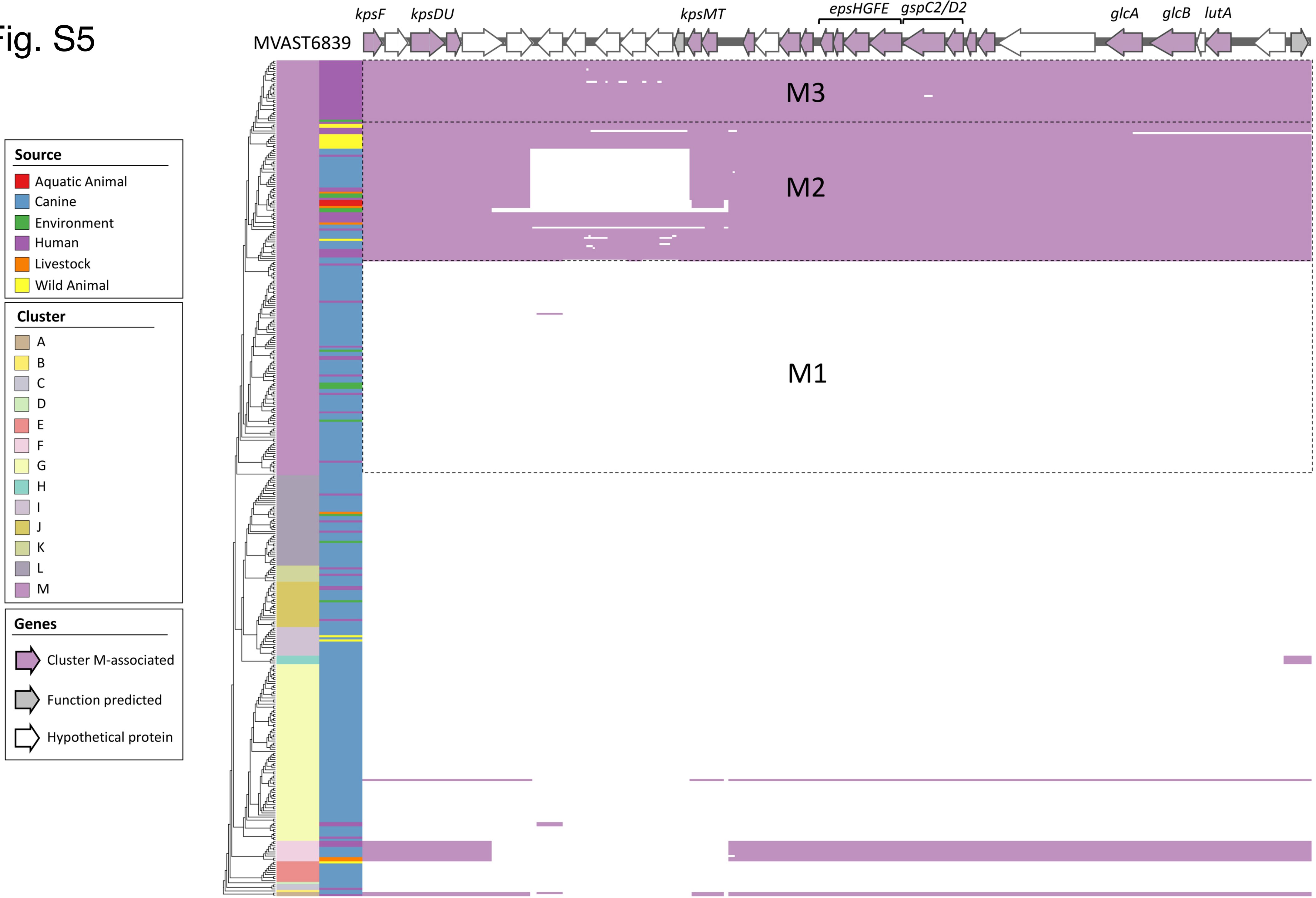

kpsF
kpsDU
kpsMT
epsHGFE
gspC2/D2
glcA
glcB
lutA
MVAST6839
M3
M2
M1
Source
Aquatic Animal
Canine
Environment
Human
Livestock
Wild Animal
Cluster
A
B
C
D
E
F
G
H
I
J
K
L
M
Genes
Cluster M-associated
Function predicted
Hypothetical protein
